# Smartphone‐based clinical diagnostics: towards democratization of evidence‐based health care

**DOI:** 10.1111/joim.12820

**Published:** 2018-09-12

**Authors:** I. Hernández‐Neuta, F. Neumann, J. Brightmeyer, T. Ba Tis, N. Madaboosi, Q. Wei, A. Ozcan, M. Nilsson

**Affiliations:** ^1^ Department of Biochemistry and Biophysics Science for Life Laboratory Stockholm University Solna SE Sweden; ^2^ Department of Chemical and Biomolecular Engineering North Carolina State University Raleigh NC USA; ^3^ Department of Materials Science and Engineering North Carolina State University Raleigh NC USA; ^4^ Electrical and Computer Engineering Department University of California Los Angeles Los Angeles CA USA

**Keywords:** Biosensors, Diagnostics, Digital pathology, Lab‐on‐a‐chip, Smartphones

## Abstract

Recent advancements in bioanalytical techniques have led to the development of novel and robust diagnostic approaches that hold promise for providing optimal patient treatment, guiding prevention programs and widening the scope of personalized medicine. However, these advanced diagnostic techniques are still complex, expensive and limited to centralized healthcare facilities or research laboratories. This significantly hinders the use of evidence‐based diagnostics for resource‐limited settings and the primary care, thus creating a gap between healthcare providers and patients, leaving these populations without access to precision and quality medicine. Smartphone‐based imaging and sensing platforms are emerging as promising alternatives for bridging this gap and decentralizing diagnostic tests offering practical features such as portability, cost‐effectiveness and connectivity. Moreover, towards simplifying and automating bioanalytical techniques, biosensors and lab‐on‐a‐chip technologies have become essential to interface and integrate these assays, bringing together the high precision and sensitivity of diagnostic techniques with the connectivity and computational power of smartphones. Here, we provide an overview of the emerging field of clinical smartphone diagnostics and its contributing technologies, as well as their wide range of areas of application, which span from haematology to digital pathology and rapid infectious disease diagnostics.

## Introduction

With the escalating use of smartphone devices in everyday life, there has been a rapid growing trend for adapting them into sensing and diagnostic needs related to medical health care 1, 2. The high level of seamless connectivity, portability and robust functionality integrated on these devices hold high promise in democratizing and decentralizing quality health care. This constitutes a solution for bridging the existing gap between healthcare professionals and patients, especially in rural areas and developing regions that are distant from centralized laboratories 3. Mobile health (m‐Health) aims to implement smartphone‐based or integrated wireless technologies to offer primary attention to these populations using audio calls, video conferences, short and multimedia messaging services or other associated applications 4, 5. One of the most enabling technologies integrated into smartphones has been the inclusion of portable digital cameras in the form of complementary metal–oxide–semiconductor (CMOS) sensors. These sensors transform electromagnetic waves within the visible spectrum into digital signals, thus enabling the capture and recording of images. Currently, the inclusion of a high definition (HD) camera is generally ubiquitous in the latest generation of smartphones, which in combination with the increasing processing and memory capacity integrated on these devices have allowed to capture digital images/photographs with high resolution.

In principle for m‐Health applications, pictures of physical signs can be captured, shared and used remotely for an initial assessment by a physician. By this, the diagnosis can be delivered quickly, and the proper encryption data can not only be efficiently recorded but also instantly shared with healthcare professionals in different locations. Mere physical signs, on most of the cases, are however, insufficient to provide an accurate diagnosis or drive a medical decision. Therefore, additional routine tests are performed at a centralized clinical laboratory to provide a specific analysis that can lead to evidence‐based decisions. This is critical for a successful treatment, management and the delivery of an integral and quality health care 6. However, most of the tests available at the clinical laboratory including microscopy, biochemistry and molecular techniques have an inherent drawback because instruments such as microscopes are commonly delicate, bulky and expensive, thus confining precise clinical diagnostics to centralized and specialized laboratories. The development of portable and integrated solutions for delivering diagnostics at the point of care (POC) has become a cornerstone to decentralize medical care. Despite a number of systems developed and commercialized in the last decade, these systems are seldom used or too expensive to be implemented at the primary care level 3, 7.

**Figure 1:**
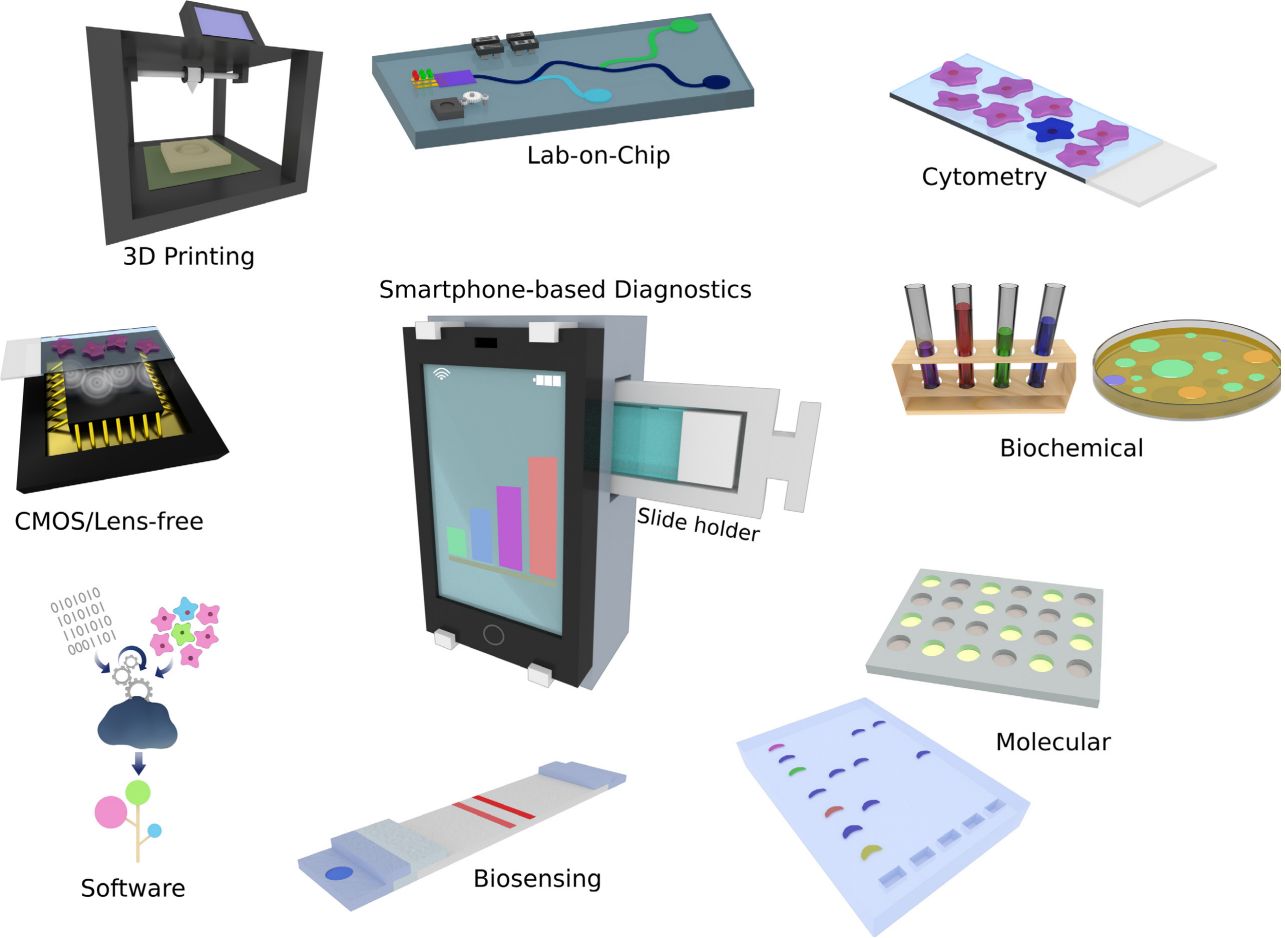
Smartphone‐based diagnostics. The use of smartphones as read‐out platforms for diagnostics has been enabled by parallel advancements in different fields including bioanalytical methods, microfluidics, biosensors and the engineering of optical attachments that interface assays with the smartphone hardware and software.

Smartphones, albeit not being designed and developed for clinical applications, can be adapted for this purpose using compatible attachments that include the necessary hardware to perform microscopic imaging, and interface with diagnostic tests integrated in lab‐on‐a‐chip devices (Fig. 1) 8, 9, 10. Parallel advancements within fields of molecular analysis, biosensors, mathematical algorithms, microfabrication, 3D‐printing and microfluidics have made possible to adapt smartphones as portable, versatile and highly connected read‐out platforms with the capability of capturing the microscopic world ranging from cells and tissues to individual DNA molecules 11, 12, 13. Smartphone‐based diagnostics is thus becoming a promising developing field that enables decentralization and democratization of clinical laboratory tests and advanced molecular techniques, making the delivery of precise diagnostics in remote areas and limited resource settings practically possible.

The aim of this review was to provide a comprehensive overview of the parallel technology advancements that have contributed to the emergence of smartphone‐based diagnostics. These include advances in bioanalytical methods, as well as technologies such as CMOS imagers, light‐emitting diodes (LEDs) and 3D‐printing techniques that are used to engineer the interfacing smartphone attachments (Fig. 1). A note on the reported applications of these state‐of‐the‐art technologies summarizes the wider scope of the platforms being developed. To finish, a critical outlook regarding this combination of disciplines and technologies comprised into the promising field of clinical smartphone imaging is provided.

## Diagnostic tests in clinical routine

Developments in the fields of analytical chemistry, molecular biology and biosensors have enabled the detection of biomarkers with an unprecedented resolution, precision and clinical relevant sensitivities. A number of techniques and methods have become available and have evolved into well‐stablished and routinely practised tests and platforms used for clinical analysis of samples such as saliva, blood, urine, stools and tissue sections. From the plethora of clinical tests available at centralized laboratories, there are a number of methods that hold a great value in improving the efficiency of health care delivery if they could be performed at POC settings 7. In general, clinical diagnostic tests can be divided in microscopy, chemistry/biochemistry and molecular based depending on the analytical technique used. Here, we provide a brief overview of the principles behind common diagnostic tests.

### Optical microscopy

Since its introduction by Kircher in the 17th century 14, optical microscopy has been established as a widely used central diagnostic tool because it allows to identify pathological changes directly on clinical samples. Furthermore, with the help of histological and microbiological staining techniques, differential observation of cellular components is possible, thus allowing to study and evaluate the integrity and identity at morphological and anatomical scales. Amongst these staining techniques, the Gram stain is commonly used for microbiology 15, Ziehl–Nielsen stain for mycobacteriology 16, Wright–Giemsa stain for haematology and cytogenetics 17 and haematoxylin and eosin stain for pathology 18. Mainly owing to their long tradition, simplicity and fast turnaround times, these microscopy techniques have stood the test of time and are ubiquitously used as gold standards at clinical laboratories in centralized and primary care facilities. In the last decade, the development of optical microscopy has been focused towards simplifying the hardware to allow for portable and battery‐powered systems with robust performance 19. Other efforts have been directed to digitalize microscopy images and automate the analysis of results. The adaption of charge‐coupled device (CCD) or CMOS‐based cameras and machine‐learning algorithms 20 has been essential developments to achieve this goal. Commercial portable solutions for brightfield and fluorescence microscopy are currently available, but it is still a challenge to provide robust handheld optical systems that allow for field applications.

### Clinical chemistry

**Figure 2:**
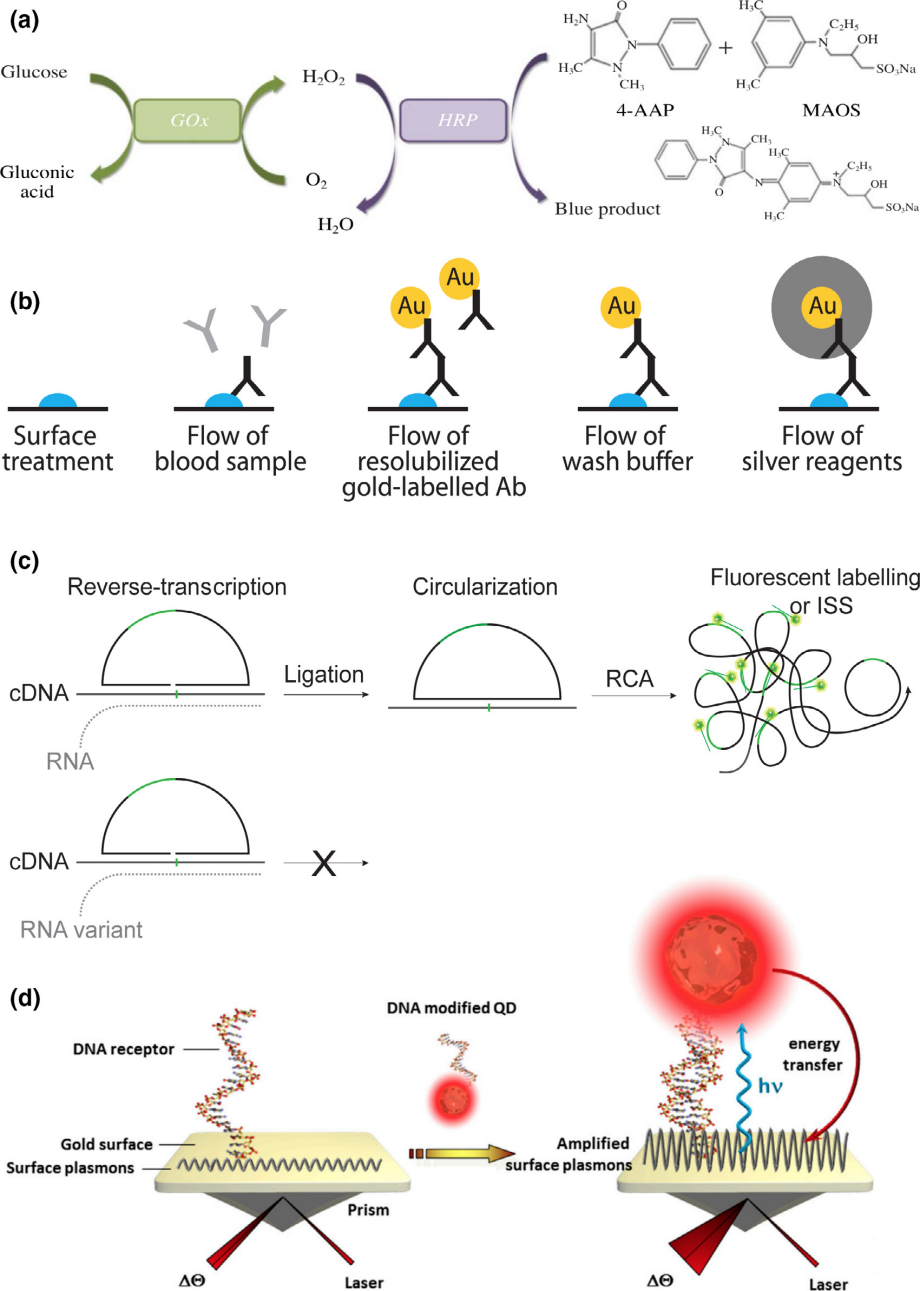
Principles of diagnostic and biosensing techniques. (a) Optical detection of glucose. Reprinted by permission from Springer Nature 104. (b) ELISA assay with colorimetric detection enhanced with AuNPs. From 125, reprinted with permission from AAAS. (c) RNA genotyping using padlock probes and RCA. Adapted from 13 CC BY 4.0 (d) DNA biosensor employing SPR and QDs. Reprinted from 168 CC BY 4.0.

Measuring the concentration of electrolytes, ions, small molecules, hormones, metabolites, proteins, drugs, complements and lipids as well as the activity of enzymes is essential for the prognosis and diagnosis of diseases such as diabetes, renal failure and heart attack amongst others 21. Generally speaking, these analyses are performed via optical or electrochemical measurements mainly in blood and urine samples. Optical methods include absorbance, fluorescence, chemiluminescence, turbidimetry and nephelometry, whilst electrochemical methods are mainly based in potentiometric measurements. The concentration of the biomarker of interest can be determined directly or by means of a specific chemical reaction using either end‐point or rate measurements. For instance, the colorimetric detection of glucose in blood is based on two catalytically induced reactions: first, glucose oxidase (GOx) reacts with glucose producing H_2_O_2,_ and second horseradish peroxidase (HRP) in the presence of the generated H_2_O_2_ acts upon a chromogenic substrate producing an apparent colour change (Fig. 2a). This is measured by quantifying the absorbance of the sample after reaction completion by means of an optical detector and correlated to the initial concentration of glucose. For diseases such as pancreatitis, the lipase activity is quantified by measuring the rate reaction. This is done by quantifying the absorbance of glycerol molecules generated over time upon lipase action on diglycerides. On the other hand, the detection of ions and electrolytes is commonly achieved with potentiometric measurements using selective electrodes or specific chemical reactions 21. These groups of biochemical tests are in general automated processes in the centralized clinical laboratory where samples are collected and batch processed routinely. Alternatively, due to their simplicity in functioning, POC tests such as the glucose test are widely available for personal use thus constituting a main approach for diabetes control.

### Molecular analysis

With the continuous advancements in the field of molecular biology, the detection of biomolecules such as DNA, RNA and proteins with sequence and single‐molecule resolution in complex clinical samples has become practically relevant and possible. Although most of the modern molecular techniques are restricted to research purposes mainly due to their high cost, specialized training and required infrastructure, a number of tests are becoming gold standards in routine clinical analysis. These include *in vitro* diagnostics (IVD) tests such as enzyme‐linked immunosorbent assay (ELISA) and polymerase chain reaction (PCR), and *in situ* analysis techniques such as immunohistochemistry (IHC) and *in situ* hybridization (ISH).

Enzyme‐linked immunosorbent assay is an extensively used IVD immunoassay to determine serum protein concentrations and the presence of viral or bacterial antigens 22. ELISA uses a combination of immobilized capture antigens or antibodies with secondary antibodies modified with a reporter enzyme. Upon target capture and recognition, sandwich immunocomplexes are formed and recognition events are identified by the activity of the reporter enzyme (Fig. 2b). Enzymes such as HRP or alkaline phosphatase are regularly conjugated to the detection antibodies, to act upon chromogenic, fluorescent or chemiluminescent substrates that generate local optical signals. ELISA is a very versatile assay that has been adapted in different formats including 96‐well plates for high throughput as well as microfluidic devices for personal testing such as the pregnancy test. Another immunoassay commonly performed is immunoprecipitation, which is based on the cross‐linking of multiple antibodies by antigen recognition in multiple sites. The cross‐linking forms insoluble complexes that precipitate allowing the detection of antigens via turbidimetry measurements. More advanced immunoassays used in clinical practice include flow cytometry, for which, a suspension of viable cells normally derived from blood or tissues are labelled with fluorescently labelled antibodies. The labelled suspension is flowed through a narrow channel and passed through a laser beam‐sensing area where individual cells are counted, classified and clustered depending on its fluorescence properties. In clinical practice, flow cytometry is used to classify and quantify immune cells for the diagnostics and characterization of diseases such as cancer and acute myeloid leukaemia 23.

Regarding nucleic acid IVD tests, PCR is the most commonly adapted technique and it is currently becoming a gold standard for viral diseases including rhinovirus, influenza and HIV 24, as well as for some genetic diseases, that is for the early detection and quantification of oncogene mutations 25. PCR relies on an exponential amplification mechanism where a nucleic acid sequence is copied up to million times during cycles of temperature changes assisted by specific combination primers and a thermostable polymerase. Using either end‐point or real‐time measurements, the presence and number of copies of a nucleic acid target sequence can be quantified with high accuracy and sensitivity. Alternatively, a number of isothermal amplification methods have been developed aiming to minimize the technical requirements for precise temperature cycling required for PCR 26. Adaption of these strategies has therefore allowed for the development of a number of POC devices 27. Commercialized systems available are mainly based on loop‐mediated isothermal amplification (LAMP), recombinase polymerase amplification (RPA) and branched DNA. The latter has been a popularly commercialized and FDA‐approved method for HIV‐1 and hepatitis C viral load testing 28.

Nucleic acid testing also offers the possibility for highly multiplexed assays that allow parallel detection and discovery of sequences, which is valuable for complex genetic diseases such as cancer. Therefore, the use of additional read‐out technologies such as microarrays and sequencing permits for higher throughput and to obtain information with higher precision and detail. Popular methods including microarray‐based IVD tests are being implemented for diagnosing diseases such as breast cancer, monitoring therapeutic response and generally genetic screening for congenital anomalies 29. Moreover, with the advent of next‐generation sequencing (NGS) technologies, the cost of nucleic acid sequencing has decreased to the point that a number of NGS‐based diagnostic assays are FDA cleared and used in highly specialized clinical laboratories, mostly for the diagnosis and characterization of cancer cases 30.

Furthermore, *in situ* methods that allow to perform immunoassays or nucleic acid detection directly on histological preparations have also become relevant for clinical practice, such is the case of IHC and ISH techniques 31. IHC makes use of antibodies conjugated to a reporter molecule, that is fluorophore or chromophore, to perform specific spatial detection of antigens directly on microbiological and histological preparations. Amongst these, immunofluorescence is considered particularly advantageous because it allows for turnaround times of less than 2 h, and has proven to be of great value in the identification of viral infections 32. The detection of specific nucleic acid sequences of interest can also be detected in a similar manner, which is achieved using a group of methods commonly referred to as ISH techniques. Similarly, ISH uses nucleic acid probes modified with chromogenic or fluorescent dyes to detect specific sequences on fixed histological specimens. The presence of the targeted nucleic acid sequences is detected and quantified by microscopy, and it can allow for multiplex analysis depending on the number of reporters and microscope filters available. In clinical practice, fluorescence *in situ* hybridization (FISH) is used for the analysis of cytogenetic abnormalities such as aberration copy number variations, duplications and translocations that are used to diagnose a number of diseases including chronic myeloid leukaemia and breast carcinomas 33. FISH has also been adapted for the detection of infectious diseases, ribosome‐mediated antibiotic resistance and parasitic diseases such as leishmania and dengue 34.

### Advanced molecular analysis with potential clinical applications

Besides these routinely used methods, there are a number of emerging molecular technologies that despite being limited to research laboratories, and in an early stage of commercialization, hold a great promise in transforming the clinical diagnostic field. Of particular interest are a number of microscopy‐based methods used for spatially resolved genomics, transcriptomics and proteomics because they allow for the quantification and analysis of biomarkers at the single cell level with an unprecedented precision and detail. The clinical validation of these methods as well as developments that allow to bring them to a clinical use is of high relevance for the development of personalized precision medicine.

In the forefront of microscopy‐based spatial analysis methods lies *in situ* sequencing (ISS), aiming to provide parallel detection of nucleic acid targets with spatial, sequence and single‐molecule resolution in preserved cells and tissues 35. Amongst these, ISS methods based on rolling circle amplification (RCA) are particularly advantageous because they require low magnification objectives. RCA is an isothermal amplification technique that locally amplifies circular DNA templates, generating long concatemeric amplicons that after FISH labelling are detected as bright diffraction‐limited spots. Circular templates can be generated with high specificity using padlock (Fig. 2c) and selector probes or by intramolecular ligation 36. RCA amplicons have been used as substrates for NGS reactions, both in microarrays and in preserved histological preparations 37, 38, 39. The high potential of this technique for clinical diagnostics has been demonstrated for tumour profiling and cell type mapping 35. Similarly, a method named proximity ligation assay (PLA), based on similar principles of ligation mediated target recognition and RCA, but for the spatial analysis of proteins has been reported potentially useful for *in situ* and *in vitro* diagnostics 40, 41.

## Enabling technologies for POC diagnostics

The development and implementation of the above‐mentioned analytical techniques have heavily relied on its integration with technologies that allowed to extend their use for clinical purposes. Towards POC diagnostics, biosensing and microfluidic platforms have permitted the development of rapid and affordable concepts based on methods that were confined to research laboratories. In this section, we provide an overview of the principles and importance of these fields for the development of portable diagnostics systems.

### Biosensors

Biosensors are analytical devices designed to detect biomarkers by transducing a biological response/interaction into a detectable output signal, for instance optical, electrical or magnetic. Biosensors are comprised of three main parts: (i) the biological recognition element for target identification, (ii) a physical transducer that converts the biorecognition event into a measurable signal and (iii) a converter that translates the signal into a readable form 42. These devices offer exceptional advantages such as ease of operation and integration, cost‐effectiveness and speed. Varied factors have contributed to the growth of the biosensor sector including advancements in biomaterials, data analytics, connectivity and mainly microfluidics 43. Furthermore, the adaption of fabrication techniques, such as screen printing for the production of enzyme electrodes, accelerated the success of biosensors as they allowed rapid fabrication and integration, thereby enabling affordable, disposable and personalized sensor devices 44. The most successful biosensing devices regarding clinical use and market share are amperometric and optical biosensors, which are the base of functioning of the glucose meters. Similarly, multiple reports describe the development of electrical and optical biosensors adapting assays such as PCR, microarrays and ELISA, for the detection of a wide range of diseases 45, 46.

Additional biosensing approaches for enhanced optical detection include quantum dots (QDs) 47 and plasmonic transducers 48. QDs are semiconducting nanocrystals and are especially suitable for effective fluorescence transduction with high multiplexing capabilities 49. Plasmonic transducers, on the other hand, are metal nanoparticles/surfaces, which are used to enhance optical responses based on the interaction of light at specific frequencies with free electrons at the metal‐dielectric interface. This phenomenon called surface plasmon resonance (SPR) can also be exploited for label‐free biosensing by detecting changes in the refractive index of a metal surface caused by interactions with attached biomolecules that affect the reflected (or transmitted) light or the resonance angle (Fig. 2d) 50, 51.

### Microfluidics

Microfluidic systems have become essential for the development of POC diagnostics because they allow consistent miniaturization, integration of complex bioanalytical assay protocols and have been of central importance for the development of biosensors. Going down to the microscale poses several superior properties including small volume requirements, small thermic mass and high surface‐to‐volume ratio, which account for decreased reagent use, shorter incubation times, parallel processing and portability 52. The rapid development of microfluidics was enabled by electronics, the fabrication of integrated circuits and the introduction of the inkjet technology in 1950 53. Microfabrication techniques further allowed the integration of sensors and microsystems allowing the introduction of microelectromechanical systems (MEMS) and the concept of micrototal analysis systems (μTAS). Nowadays, they are widely used as a versatile platform for biomolecule analysis and diagnostics 54, 55, 56.

Microfluidic devices can be manufactured using a variety of materials including glass, silicon, thermoplastic polymers and paper 57. For POC applications, microfluidic paper‐based analytical devices (μPAD) are attractive due to properties such as low cost, mass producibility, disposability and ease of operation. These properties render them ideal for the development of portable and power‐free diagnostic platforms, thus standing out when compared to classical μTAS. Commercially available μPADs are in the format of dipsticks and lateral flow assays (LFA) with a wide range of applications from small analytes in blood, such as ions and sugars, to cells in urine and detection of proteins and nucleic acids.

Recently, 3D printing has become an emerging technology that holds promise of being production scalable, cost‐effective as well as rapid, versatile for manufacturing custom‐made designs and offering high precision, thus rendering it as a beneficial technology for the production and low‐cost prototyping of POC devices 58. Examples include miniaturization of instrumentation for low‐cost qPCR, fluorescent microscopy and finger‐powered microfluidic pumps 59. Furthermore, approaches such as free and open‐source blueprints for laboratory equipment further push the 3D printing technology revolution and microfluidic field for automation and miniaturization of bioanalytical techniques 60.

## Smartphone‐based imaging technologies

From the read‐out point of view, except for those tests that can be read by naked eye, most of the described platforms and technologies rely on optical or electrical detectors that require either specialized instrumentation, expensive hardware components or computational power to acquire, analyse and deliver data. Smartphones have evolved into powerful portable gadgets that integrate high processing power, together with physical sensors and connectivity. The image sensors within the smartphone's camera module are sensitive enough for many diagnostically relevant applications. Details outlined below highlight the recent progress in developing various optomechanical attachments to turn smartphones into robust read‐out devices for diagnostics without permanently altering the phone. Most of these devices have also been designed to limit manufacturing costs which is essential in lowering the barrier between low resource settings and modern medical techniques.

### Brightfield microscopy

**Figure 3:**
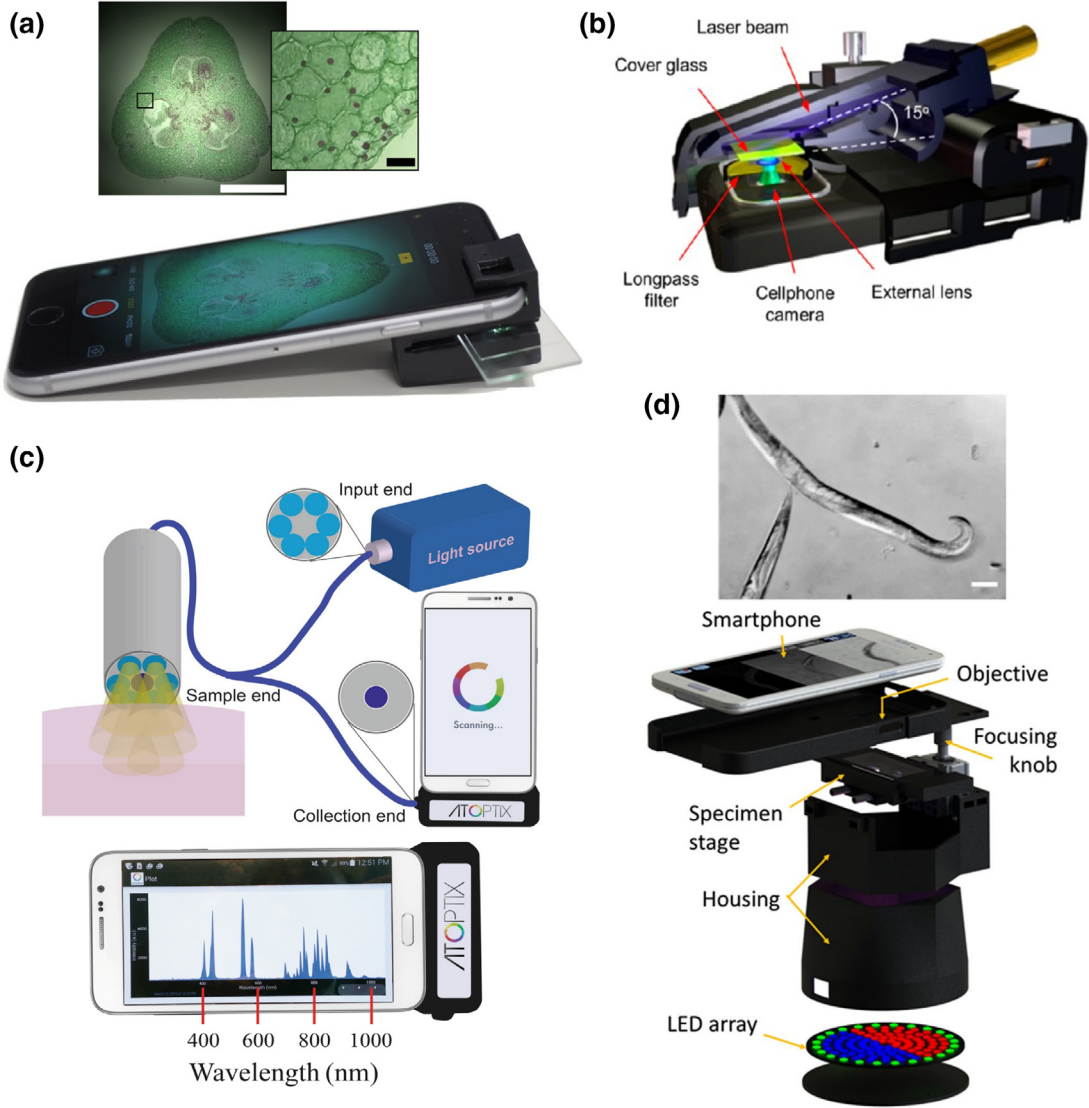
Smartphone based imaging technologies. (a) Smartphone attachment for brightfield and darkfield imaging. Scale bars inset images: 1 mm and 50 μm respectively. Reprinted from 63 CC BY 4.0. (b) Components to achieve fluorescence microscopy with a smartphone. Reprinted with permission from 73. Copyright 2013, American Chemical Society. (c) A smartphone spectrometer configuration with optical fiber and micro USB interface for operation. Reprinted from 169. CC BY 4.0. (d) Configuration of a multi‐contrast smartphone microscope with color‐coded LED illumination patterns. Reprinted from 84 CC BY 4.0.

Smartphone cameras use low‐cost CMOS technology, which are able to detect red, green and blue (RGB) light and therefore ideal for optical quantification in the visible wavelength range. An initial application of the integrated CMOS sensors in smartphones was to use them as external cameras for capturing images from the microscope ocular using 3D‐printed adapters 61, 62. Furthermore, multiple studies have reported the design and development of attachments that are designed to comprise simplified optics that allow to perform microscopy observations directly 63. Perhaps the most unassuming yet most helpful of these applications is the smartphone brightfield microscope, which can be formed in a simple configuration by adding an external lens in front of the smartphone camera (Fig. 3a). One method described the adaptation of a smartphone into a usable microscope by mounting a 1‐ or 3‐mm ball lens directly to the phone's camera 64, 65. The authors were able to achieve a resolution of 1.5–10 μm with a field of view (FOV) of approximately 150 × 150 μm without postprocessing. The main disadvantage to this approach was the distortion around the edge of the images due to the curved nature of the ball type lens.

A query of the correlation of resolution to pixel density in smartphone image sensors was performed by affixing an objective lens and eyepiece to a smartphone 66. The study surveyed iPhone and Android phones released between 2007 and 2012 and demonstrated that using a smartphone equipped with an 8 MP sensor and 40×/NA 0.65 objective, a spatial resolution of ~0.87 μm could be achieved whilst sacrificing diagnostically relevant FOVs. To maintain sufficient spatial resolution and large FOV at the same time, a camera lens from a smartphone can be reverse‐mounted to another smartphone 12, 67. With this design, a spatial resolution of ~5 μm with a FOV of 10 mm^2^ can be achieved. By further trading off some of the resolution, the authors were able to increase the effective FOV to 15.7 mm^2^ whilst still keeping the spatial resolution below 10 μm.

Perhaps the least complicated and most cost‐effective method for converting a smartphone into a brightfield microscope is the inkjet printing of lenses using (polydimethylsiloxane) PDMS 68. Researchers were able to produce small lenses that can be placed directly in front of the smartphones camera lens. A spatial resolution of 1 μm with a total magnification of 120× was reported whilst the estimated cost per lens was ~$0.01 68.

Finally, some recent results have also demonstrated the use of machine‐learning techniques, in particular deep neural networks, to enhance mobile microscopy images by correcting the spatial and spectral aberrations in the raw acquired images of mobile phone‐based microscopes 69, 70. These recent efforts on the use of deep learning to transform images and enhance the spatial resolution of a microscopy system would significantly benefit low‐cost and mobile microscopy systems, by closing the gap between the performance of mobile microscopes and the state‐of‐the‐art benchtop imaging systems.

### Fluorescence microscopy

Fluorescent microscopy is essential for modern biomedical diagnostics. To this application, smartphones have proven themselves to be more than capable of delivering diagnostically relevant results when equipped with the proper attachments. A typical smartphone fluorescence microscope consists of an excitation light source (LED or laser diode) and an emission filter, in addition to the above‐mentioned external lenses with the brightfield modality. How the samples are illuminated is the key consideration when designing a smartphone‐based fluorescence microscope. For samples such as cuvettes or tubes, an orthogonal illumination to the detection path provides a simple means of high signal‐to‐noise ratio (SNR) fluorescence detection 71. For planar sample slides, waveguide coupling from the side of the glass substrate or from the end of a glass capillary tube has been proven to be an efficient background rejection method for sensitive fluorescence imaging 72. An alternative approach is the tilted illumination of the sample slides to achieve high SNR and therefore improved detection sensitivity (Fig. 3b). In this configuration, the samples are back‐illuminated by the excitation beam from a compact laser diode at an incidence angle significantly larger than the numerical aperture of the external lens. This design enables the capture of fluorescent signals from a few hundred fluorophores within a diffraction‐limited spot using the CMOS sensor, offering opportunities to visualize single 100 nm nanoparticles, individual virus particles and single DNA molecules 12, 13, 73.

Due to the small pixel sizes and larger operating temperature, smartphone devices are in general not as sensitive to light signals as their commercial counterparts. However, to enhance the fluorescence detection the use of glass capillary tubes as sample containers are useful for both reducing sample volumes and improving detection sensitivity 74, 75. Plasmonics can also be used to enhance the signal intensity of smartphone fluorescence microscopy enabling the detection of single 50 nm fluorescent beads and individual QDs (ca. 20 nm dia.) with a smartphone 76. The sensitivity limit of this platform was estimated to be around 80 fluorophores per diffraction‐limited spot by imaging DNA origami‐based brightness standards labelled with different numbers of fluorophores.

Some approaches are currently investigated to mitigate the limiting SNR that is typical of smartphone image sensors error, for instance, a radiometric method where two different wavelengths corresponding to the analyte were measured simultaneously instead of the typical one. By observing the correlation of the two wavelengths false‐positive and false‐negative signals could be discarded with more accuracy 77.

### Spectroscopy

Smartphone‐based spectroscopy has seen much more attention as image sensor quality continues to improve. The high precision requirements of spectroscopic methods have been prohibitive in the past but as smartphones continue to get more sophisticated so do the applications for which they qualify. The optical design to achieve spectrophotometric measurements is relatively simple (Fig. 3c). An incident light source is shown through a pinhole which is then collimated and polarized. The light then passes through the sample and is refocused onto the diffraction grating through a cylindrical lens, to direct it to the CMOS sensor. Recently, the construction of a smartphone‐based spectrometer was reported using a DVD diffraction grating system. The device was able to sample the entire visible spectrum with a high spectral resolution decreasing the cost significantly from $75 for a commercial system to about 2.5 cents 78.

Background illumination is always a challenge when designing spectroscopic systems. This problem exacerbates when the system must be portable and easily attached to a smartphone. This was addressed in a study using a fibre‐optic bundle to direct light from the smartphones flash directly onto the sample 79. The light from the sample was then delivered by an endoscopic fibre bundle for diffraction grating. From this point, the light was focused using a cylindrical lens with a focal distance of 2.0 cm onto the CMOS image sensor of the smartphone. This platform was able to measure a bandwidth of ~250 nm with a spectral resolution as low as ~2.0 nm. The use of fibre‐optic bundles to guide the incident light is becoming more prevalent as researchers recognize its efficiency in eliminating noise from background illumination.

### Phase imaging

Phase imaging is a desirable capability of portable imaging devices because it allows the analysis of transparent samples without staining or dyeing 8, 9. Moreover, typical phase imaging requires bulky equipment and experienced operators to use. There have been various demonstrations of phase imaging in compact and cost‐effective embodiments, targeting POC settings 80. As a recent example, researchers integrated a quantitative phase imaging method for blood testing using a smartphone 81. This system combined with an image processing algorithm enabled a resolution of about 1 μm as well as to construct accurate 3D phase maps. Other work reported a quantitative phase microscope using a 60× micro‐objective connected to the smartphone camera lens by an eyepiece, and using a LED light source and the manual camera focusing capability 82.

Smartphone‐based differential phase contrast (DPC) microscopy can also be achieved in a single‐shot image by asymmetric illumination patterns using LED arrays. Images are reconstructed from two sequential images acquired with complementary illumination patterns which in this case are using half of the LEDs 83. Furthermore, LEDs with different colours can be used to generate colour‐encoded illumination patterns to avoid synchronization between image acquisition and pattern illumination 84. As a result, DPC can be readily generated in a single‐shot smartphone image by decomposition of the RGB channels and computational reconstruction of the different colour channels (Fig. 3d).

### Light scattering

Light scattering is an essential method for noninvasive analysis of suspended particles in solution. As such, the adaptation of smartphones into light scatter sensors can be utilized to perform on‐site medical diagnosis. The viability of the smartphone platform for scattering‐based sensing was examined using simple optical elements and a laser to determine the mean size of suspended particles within a 20 nm accuracy 85. This application was revisited later using a relatively complex optical array contained within a compact housing 86. The authors were able to utilize the phone flash LED as incident light which was reflected onto a perfluorinated prism in contact with the sample solution housed in a cuvette. From here the reflected light from the imaging surface passes through a polarizer, a converging lens and then a final converging lens before it contacts the smartphone image sensor.

## Smartphone‐based diagnostic systems

The adaption of smartphones as imaging read‐out platforms in the clinic could be used for on‐site data acquisition, analysis in a real‐time mode, management of the generated information at the convenience of the user and enormously fast transfer of data from the site of detection to healthcare professionals. Before the last decade, clinical analytical methods and smartphone technologies were independent disciplines; in this section, we include a review of the smartphone‐based imaging systems that combine these disciplines with diagnostic purposes. Table 1 summarizes a number of representative papers listing the targeted diseases and the related clinical areas of application.

**Table 1:** Summary of the applications of smartphone‐based diagnostics and sensing

Disease/Pathology *(Discipline)*	Biomarker	Sample	Detection mode	Comments	Ref.
Malaria *(Infectious disease diagnostics)*	*Plasmodium falciparum*	Whole blood	Brightfield	Sensitivity = 90% Specificity = 90%	161
Cystic fibrosis, emphysema *(Pulmonology)*	Secretory leucocyte protease inhibitor	–	Electrochemical (Potentiometry)	LOD = 1 nmol L^−1^	160
Dialysis‐related amyloidosis *(Nephrology)*	β_2_‐Microglobulin	–	SPR	LOD = 0.1 μg/mL	156
AIDS, hepatitis and flu *(Virology)*	HIV Hepatitis B and C Influenza	Whole blood, serum and plasma	Fluorescence (QDs)	LOD = 10^3^ copies per mL	154
Gastroenteritis *(Microbiology)*	*E. coli* O157:H7	fat‐free milk	Fluorescence (QDs)	LOD = 5–10 cfu mL^−1^	152
Giardiasis *(Infectious disease diagnostics)*	*Giardia cysts*	Water	Fluorescence	~1 cfu mL^−1^	20
Skin cancer *(Oncology)*	Kaposi's sarcoma herpesvirus	Skin biopsy	Fluorescence (Thermal PCR)	Assay time = 30 min	151, 163, 164
Anaemia, leukaemia *(Haematology)*	WBC, RBC counts	Blood	Fluorescence and brightfield	<10% error in cell densities	165
Anaemia, leukaemia *(Haematology)*	Blood types/haematocrit level	Blood	Brightfield	15% prediluted blood; 3 μL volume	147
Hormone profiles *(Endocrinology, gynaecology)*	Pregnanediol glucuronide	Urine	Colorimetric	Accuracy = 82.20%	112
Stress, anxiety and depression *(Endocrinology, psychiatry)*	Cortisol	Saliva	Chemiluminescence	LOD = 0.3 ng mL^−1^	114
AIDS, flu and haemorrhagic fever *(Virology)*	HIV‐1‐p17 hemagglutinin dengue virus type I	Plasma blood	Bioluminescence	LOD = 100 pM	153, 166
Haemorrhagic fevers *(Infectious disease diagnostics)*	Zika Chikungunya, dengue viruses	Blood Urine Saliva	Fluorescence	LOD = 22 PFU mL^−1^	140
Pulmonary tuberculosis (Infectious disease diagnostics)	*Mycobacterium tuberculosis*	–	Colorimetric (AuNP‐enhanced)	time = 65 min LOD = 10 μg mL^−1^	167
Herpes *(Virology)*	Herpesvirus	–	Colorimetric (AuNPs‐enhanced)	LOD = 5 nmol L^−1^	151
Prostate cancer *(Oncology)*	Prostate‐specific antigen (PSA)	Whole blood	Colorimetric Fluorescence	Colorimetric detection: Assay time = 13 min LOD = 0.4 ng mL^−1^ Fluorescence detection Assay time = 22 min LOD = 0.08 ng mL^−1^	137
Colorectal cancer *(Digital oncology)*	Oncogene *KRAS* mutations	Tumour tissue sections	Fluorescence (Targeted ISS)	LOD: 1, fM; 1 : 1000, mutant: wild type ratio	13
Mild traumatic brain injury *(Neurology)*	Brain‐derived exosomes	Serum	Fluorescence	Assay time = 1 h LOD = 107 exosomes per mL	133

### Light microscopy applications

In a study by Hutchison *et al*. 87, the detection of anthrax was enabled via a smartphone‐based brightfield microscope that was used to monitor the growth of *Bacillus anthracis* spores on a microfluidic incubation chip. This system was capable of detecting from 50 to 5000 spores in a period of 3–5 h. Furthermore, field tests in rural Ghana and Côte d'Ivoire were conducted to evaluate the performance of a smartphone‐based brightfield microscope 88 as well as CellScope, a compact smartphone‐based microscope with the Newton Nm1 microscope, a commercial portable microscope 89. Both systems showed a performance comparable to a conventional microscope to detect *Schistosoma mansoni* and *S. haematobium* eggs in stool and stained urine samples 88, 89. Similarly, a reversed‐lens CellScope and a smartphone‐mounted Foldscope have been demonstrated for the detection of helminth eggs in stool and urine samples 90, 91, 92. CellScope has also been employed to detect *Loa loa* filariasis on whole blood thick smears of 300 samples achieving a specificity and sensitivity of 94% and 100%, respectively 93.

Moreover, malaria parasites, sickled blood cells and tuberculosis (TB) bacilli have been captured with a high resolution with smartphone microscopes that include brightfield and fluorescence in blood and sputum samples 94, 95, 96. Cell counting and cytology applications are also possible using a regular smartphone‐based imaging system combined with a microfluidic chip with surface functionalization for specific capture and counting of CD4‐positive T cells 97, 98. Other cost‐effective mobile imaging systems were also developed for imaging and detection of CD4‐ and CD8‐positive cells 99.

### Clinical chemistry applications

**Figure 4:**
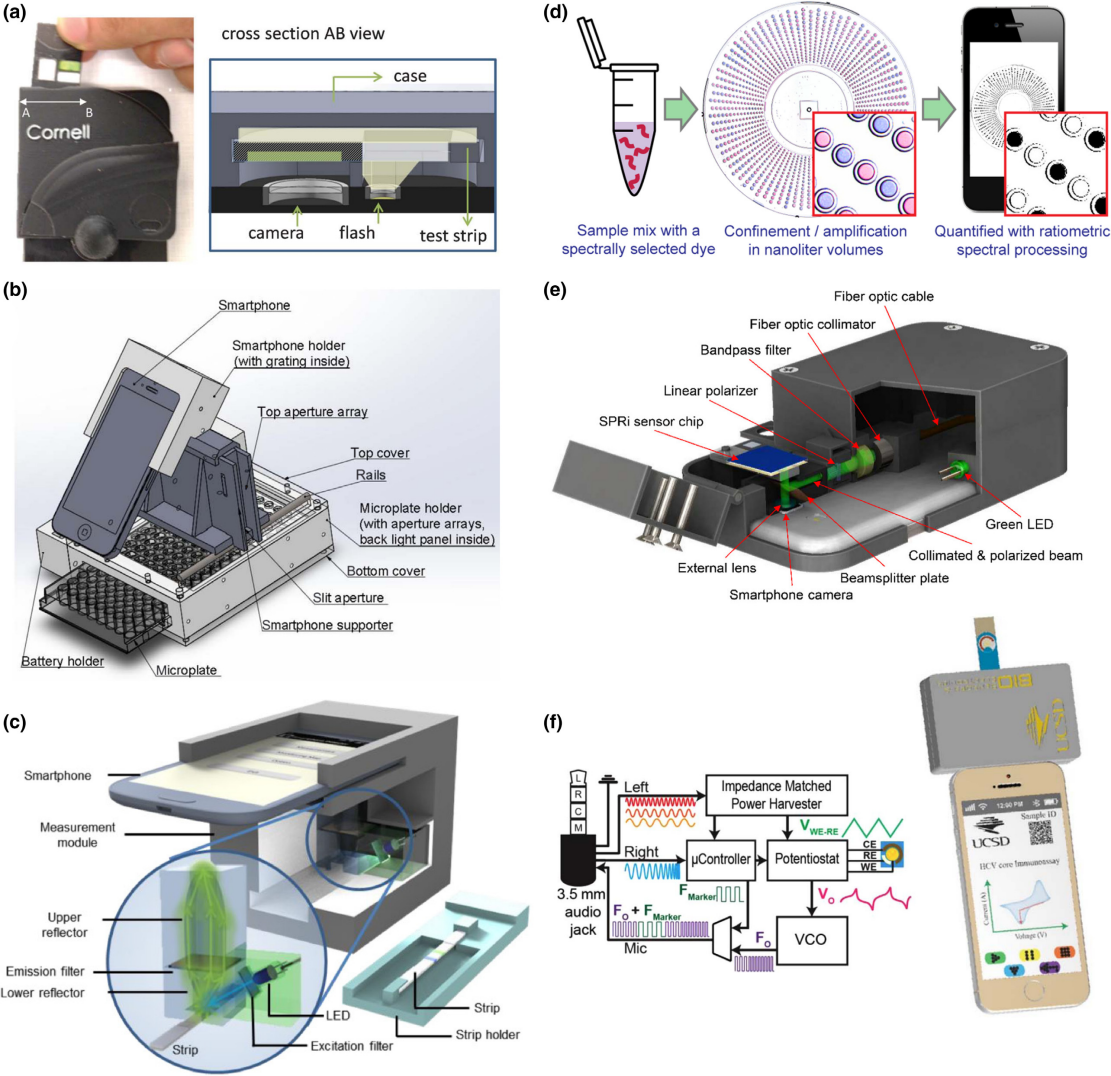
Examples of smartphone based diagnostic systems. (a) Smartphone accessory for colorimetric detection of pH in sweat and saliva. Reprinted from 107 by permission The Royal Society of Chemistry. (b) High throughput smartphone spectrophotometer for cancer diagnostics by detection of IL‐6. Reprinted from 123 with permission from Elsevier (c) Fluorescent LFA strip smartphone reader for POC influenza diagnostics. Reprinted from 127 CC BY 4.0 (d) SlipChip‐based digital single‐molecule LAMP with a smartphone read‐out for HCV detection 142. (e) SPR imaging with a smartphone for detection of IgG. Reprinted from 158 with permission from Elsevier (f) Smartphone‐based electrochemical sensing for HCV and SPLI harvesting power through the headphone port. Reprinted from 170 with permission from Elsevier.

The high demand and relevancy of routine chemistry tests in body fluids have led to the development of many smartphone‐based diagnostic tools. For instance, several devices were designed to rapidly measure the levels of glucose in whole blood, serum and urine samples using microfluidic chips and smartphone‐based colorimetric readers 100, 101, 102, 103, 104. Similarly, the concentration of lactate in oral fluids and sweat samples have been measured via HRP‐induced colorimetric and chemiluminescent reactions coupled to smartphones 105, 106, as well as sensitive measurements of pH and Na^+^ ions in sweat and saliva samples (Fig. 4a) 107, 108. For instance, for the detection of Na^+^ ions, Lipowicz *et al*. 108 adopted a sample microfluidic chamber with a special geometrical shape and a holographic diffraction grating film, which allowed to focus the excitation light and hence improve the fluorescence signals from the samples. Similar approaches have been reported for electrolytes such as potassium, chloride and calcium 109, 110.

Additional colorimetric and fluorescence smartphone‐based readers have also been developed for detecting cholesterol, steroid hormone, protein, cortisol, thrombin and sperm 111, 112, 113, 114, 115, 116. Jalal and colleagues, for example, developed a disposable μPAD device to detect simultaneously glucose, protein, pH and red blood cells in urine samples with a smartphone 117. Another reported platform included the concentration measurement of glutamate in clinical samples to diagnose neurodegenerative disorders 94. Similarly, Christodouleas *et al*. 118 used a smartphone camera as a photometer to simultaneously measure the levels of lactic acid, low‐density lipoprotein, antitreponema pallidum antibodies, total protein, haemoglobin and nitrite ions in various sample matrices. Finally, urinary tract infections can be diagnosed by means of a smartphone reader by measuring the metabolic activity of bacteria cultured in a microwell array with a colour indicator. This system has reported a range of detection from 10 to 10^6^ cfu mL^−1^ 119, 120.

### Immunoassays

Combining molecular tests with smartphone readers with the help of microfluidics and biosensors has allowed the development of several POC concepts for various diseases. For example, a cost‐effective and compact cellphone‐based LFA reader platform that works with various lateral flow immunochromatographic assays was created and tested with malaria, TB and HIV LFAs by installing it on both Android‐based smart phones as well as an iPhone 9. As another example, Wang and colleagues designed a platform termed HiSOP capable of simultaneously screen 64 samples with ELISA using a 3D‐printed microprism array and a smartphone. This array was implemented to overcome the small FOV of the smartphone's camera. The accuracy of HiSOP was evaluated by performing multiplex ELISA of human interleukin 6 (IL‐6) protein and six types of plant viruses 121. The measurement of IL‐6 and Ara h1 (a peanut allergen) levels has also been reported with a microplate ELISA assay coupled to smartphone‐based spectrometer (Fig. 4b) 122, 123. Similarly, microfluidic ELISA chips have also been combined with smartphones for detecting sexually transmitted diseases 124, 125. In addition, Joh *et al*. 126 developed a portable platform using inkjet‐printing technology to produce microarrays with enhanced binding properties for sensitive sandwich immunoassay. This platform was demonstrated for markers related to prostate cancer, endocrinology, cytokine profiling, cardiology and HIV 126.

Diagnosis of viral diseases such as avian influenza has been reported with a sensitivity of 96.5% and specificity of 98.5% using sandwich ELISA integrated into fluorescent LFA strips and imaged with a smartphone (Fig. 4c) 127. Additionally, the multiplex colorimetric detection of herpes simplex virus types 1 and 2 (HSV‐1 and HSV‐2), as well as mumps and measles viruses was achieved using an array of optical fibres that allow to screen 96 microwells with a smartphone 128. For detecting hepatitis C virus (HCV) in serum, an immunochromatographic microfluidic device comprised of microchannels filled with a hydrogel with patterned immobilized antigens has been used. Upon recognition of HCV and addition of fluorescently labelled secondary antibodies, the barcode‐like patterns are imaged and analysed using a smartphone 129.

Additional smartphone‐based platforms have been developed to target conditions such as kidney damage, heart failure, sickle cell anaemia and osteoarthritis 71, 130, 131, 132. For example, Ko and colleagues designed a portable diagnostic platform consisting of a microfluidic device and smartphone‐based fluorescence detector to profile brain‐derived exosomes in serum samples 133. Using similar approaches, the concentration of human C‐reactive protein (CRP) has been possible 134, 135. Moreover, ovarian cancer biomarkers in urine samples were detected with a total assay time of 5 h and an LOD of 19.5 ng mL^−1^ using a microfluidic ELISA format and a smartphone to image and analyse the results 136. The detection of prostate‐specific antigen (PSA) has also been achieved by similar means 69. The platform, termed ‘MCFPhone’, was capable of detecting PSA with brightfield and fluorescence modes resulting in LODs of 0.4 and 0.08 ng mL^−1^, respectively 137.

### Nucleic acid detection

Nucleic acid amplification techniques such as PCR, RCA and LAMP have been integrated into portable smartphone‐based devices. For example, Stedtfeld and colleagues developed a microfluidic device interfaced and operated with an iPod Touch for the simultaneous detection of *Escherichia coli*,* Staphylococcus aureus*, virulence and resistance markers. The microfluidic device integrated a LAMP assay with fluorescence labelling, reporting the detection of down to 30 target copies 138. Similarly, POC herpes diagnosis has been achieved using a ‘smart cup’ format. The smart cup was used as the microfluidic chip housing and to isolate and regulate the heat required for the LAMP reaction, and the lid was designed to attach an android smartphone for real‐time fluorescence quantitative detection of HSV‐2 139. Additional studies report the detection of viruses such as HIV, zika, chikungunya and dengue, using reverse‐transcription LAMP (RT‐LAMP) integrated into smartphone‐microfluidic POC systems and demonstrated with urine, blood and saliva samples 140, 141. Furthermore, a digital RT‐LAMP assay for single‐molecule HCV detection using SlipChip microfluidics was demonstrated in clinical and test samples (Fig. 3d). The study compared the performance of different smartphones for image acquisition 142.

Some studies have reported simplified approaches to perform PCR in devices that can be interfaced with smartphones and allow portability. For instance, a convective PCR assay for amplifying hepatitis B virus (HBV) DNA without a thermocycler was adapted to a smartphone read‐out. The study reports a clinical evaluation study with 60 samples resulting in assay accuracies of up to 100% 143. Other approach developed a solar thermal PCR system that allowed to perform sunlight‐driven DNA amplification under a range of ambient conditions for detecting Kaposi's sarcoma herpesvirus (KHSV) in human skin biopsies 144. Other smartphone‐based genotyping platforms include for instance a microarray decoding platform designed to detect nine mutations associated with hereditary hearing loss using a smartphone with a 3D‐printed optical attachment for imaging and recording the microarray results 145. Moreover, an RCA‐based microarray for genotyping of liver cancer‐associated mutations with exhaled breath condensates was achieved. More specifically, the wettability of the detection regions on the glass substrate changes as a result of the formation of RCA amplicons, this allows for a visual read‐out based on the contrast between the hydrophilic and hydrophobic regions that can be recorded with a smartphone camera 146. Furthermore, as a step towards portable sequencers, Kühnemund and colleagues reported a smartphone‐based multimodal microscope that is capable of imaging single RCA amplicons for *in situ* genotyping and targeted ISS. The system was composed of a 3D‐printed attachment for fluorescence and bright‐field imaging as well as a 3D stage for flexible positioning of microscope slides. Tumour tissue sections and cell DNA extracts were analysed for sequencing and detecting mutations related to cancer 13.

### Biosensing applications

Smartphones are equipped with advanced physical sensors including gyroscopes and magnetoscopes. Multiple concepts are being developed to equip smartphones with biosensing capabilities exploiting the advantages of combining established biosensing platforms with the inherent properties of smartphones including analytics, portability and connectivity. To this end, optical paper‐based biosensors are in particular advantageous because the interfaces can be quite simple. Barcode‐like paper sensors with developed apps for image acquisition and analysis have been demonstrated for applications such as blood typing haematocrit level determination, glucose and uric acid measurements 147, 148. Multilayer‐modified paper substrates have been adapted with a smartphone read‐out for the detection of L‐lactate in oral fluid and tears 105.

Other optical smartphone biosensing platforms based on the SPR properties of gold nanoparticles (AuNPs) have been proposed as diagnostic tools 98. For instance, the concentration of vitamins such as B12 and D in blood and serum samples has been measured by performing an AuNP‐based immunoassay along with a smartphone‐based colorimetric reader 149, 150. Other applications include the diagnosis of KSHV, via a colorimetric assay based on the aggregation of functionalized AuNPs in the presence of the viral target DNA 144. The aggregation causes a strong enhanced colour change in the particle suspension allowing to use simple smartphone detector to perform the analysis with high sensitivity 151.

Additional approaches have reported the detection of *E. coli* O157:H7 down to 10 CFU μL^−1^ with a smartphone using QDs and capillary arrays 152. Moreover, Arts and colleagues developed a protein sensor to detect HIV and dengue using a smartphone as the sole piece of equipment 153. QDs have also been adapted in combination with a paper‐in‐PDMS chip to achieve the single‐step detection of thrombin 115. Ming *et al*. 154 implemented QD barcodes to detect HVB and HIV in clinical samples using LAMP, obtaining a linear range of detection from 10^3^ to 10^9^ copies mL^−1^. The sensitivity of the fluorescence resonance energy transfer (FRET) between QDs donors and Cy3 acceptors was also exploited as a means to perform radiometric sensing of nucleic acid hybridization events on a μPAD platform 155.

Label‐free smartphone biosensors approaches mainly based on SPR measurement detection for protein detection are also available. Actual smartphones screens provide more than sufficient wide‐angle illumination to perform SPR measurements. For example, using a screen side optical attachment, it was possible to detect human β_2_ microglobulin with a LOD of 0.1 μg mL^−1^ using a commercial SPR sensor chip 156. Another study showed the application of fibre‐optic‐based SPR as a key approach to eliminate background illumination in smartphone detectors 157. More recently, attempts to reduce the cost of SPR imaging was demonstrated using a blue ray disc coated in a silver/gold bilayer structure to perform the plasmonic imaging (Fig. 4e) 158. Alternatively, a label‐free spectroscopic biosensor to measure immunoglobulin G (IgG) concentration using photonic crystals has recently been demonstrated 159.

Finally, electrochemical biosensors have also been used in combination with smartphones. Examples include a potentiostat for monitoring lung infections in cystic fibrosis patients by tracking the concentration of secretory leucocyte protease inhibitor. This was achieved using an electrochemical ELISA platform that is plugged into the audio port of the smartphone (Fig. 4f) 160. Fraser *et al*. developed a portable and equipment‐free biosensor for colorimetric detection of the *Plasmodium falciparum* lactate dehydrogenase enzyme for malaria diagnostics. They reported 90% sensitivity and specificity for clinical samples using their three‐chamber microfluidic chip in combination with automated smartphone read‐out 161.

## Conclusions and outlook

Smartphone‐based diagnostics is a powerful approach that could strongly influence how medicine is currently being practised, probably and hopefully in a similar manner as how smartphones revolutionized communications. The implementation of these systems into the medical practice could not only allow the decentralization of medical specialties such as oncology, haematology and virology, but also allow for fast disease data acquisition, storage and management. This could have a significant impact for epidemiology and public health because attached to the diagnostic test result, accurate geographic and demographic data can be stored. Furthermore, exploring and understanding the limitations regarding the viability of these platforms for field use are going to be essential for moving forward towards bedside diagnostics and democratization. For a system to be truly universal, it must be able to be affordable for those with very limited means; therefore, an important direction of research in any of these methods will be further reduction in the costs. This is especially the case for advanced molecular methods that require expensive reagents and special storage/transport conditions. Commercialization approaches that invest on increasing the scalability and robustness of the assay/test are essential to achieve cost‐effective and robust platforms designed for limiting and harsh conditions. Furthermore, assay integration plays a central role in this regard to be able to deliver platforms that can be interfaced with both the optical and electrical components of smartphones.

An important consideration is the availability of off‐the‐shelf smartphone components that would allow their use in a modular fashion. Initiatives like fair phone (www.fairphone.com) or the shelved project ARA will be crucial to enable scalable manufacturing of smartphone technology‐based diagnostic devices. For instance, CMOS sensors can be used to build lens‐free microscopy systems that hold promise for on‐site diagnostics because they allow for wide FOVs, which could be relevant for infectious diseases such as malaria and TB 162.

The studies mentioned in this review demonstrate that sensitive and specific bioanalytical assays are already available in a number of formats and at different levels of integration with smartphones. However, most of the reported approaches are in a proof‐of‐concept phase or constitute isolated efforts, and just a few are in commercialization or productization stage. Further focus is to be laid on validating these platforms and assessing their feasibility in clinical settings. In the near future, it is certain that smartphone‐based diagnostics would strike the needed balance between molecular methods and compatible technologies for bedside diagnostics, thus becoming a central part of the m‐Health ecosystem, and ultimately democratizing evidence‐based medicine.

## Conflict of Interest

A.O. reports grants from NSF during the conduct of the study and other from Holomic LLC, outside the submitted work; in addition, A.O. has several pending patents on mobile diagnostics and imaging systems with royalties paid. The remaining authors have nothing to disclose.
